# Cognitive deficits in children with autism spectrum disorders: Toward an integrative approach combining social and non-social cognition

**DOI:** 10.3389/fpsyt.2022.917121

**Published:** 2022-08-08

**Authors:** Melek Hajri, Zeineb Abbes, Houda Ben Yahia, Selima Jelili, Soumeyya Halayem, Ali Mrabet, Asma Bouden

**Affiliations:** ^1^Razi Hospital Child and Adolescent Psychiatry Department, Faculty of Medicine of Tunis, Tunis El Manar University, Manouba, Tunisia; ^2^Razi Hospital Child and Adolescent Psychiatry Department, Manouba, Tunisia; ^3^Health Minsitery, General Directorate of Military Health, Faculty of Medicine of Tunis, Tunis El Manar University, Tunis, Tunisia

**Keywords:** autism spectrum disorder, children, cognitive remediation, executive functions, social cognition, Cognitive Remediation Therapy

## Abstract

Autism spectrum disorder (ASD) is associated with neurocognitive impairment, including executive dysfunctioning and social cognition (SC) deficits. Cognitive remediation (CR) is a behavioral training-based intervention aiming to improve cognitive processes. Its first use in psychiatry interested patients with schizophrenia, in whom promising results have been shown. Integrated CR programs targeting both social and non-social cognition have demonstrated to be effective in improving both cognitive domains and functional outcomes. CR studies in children and adolescents with ASD are still new, those regarding CR approaches combining social and executive functioning remediation are scares. One study examining the efficacy of cognitive enhancement therapy (CET) for improving cognitive abilities in ADS adults, showed significant differential increases in neurocognitive function and large social-cognitive improvements. Therefore, taking into account the overlap between ASD and schizophrenia, and considering the close link between executive functions (EF) and SC, we suggest that integrative approach in ASD could result in better outcomes. The present perspective aimed to highlight cognitive remediation (CR) programs contributions in ASD (especially in children and adolescents), and to discuss the value of combining social and non-social programs.

## Introduction

Since Kanner's first description, interest devoted to autism has been constantly growing (1). Currently known as autism spectrum disorder (ASD) according to DSM-5, it is a lifelong neurodevelopmental disorder characterized by difficulties in social interaction and communication, as well as restricted and repetitive behavior and interests (2).

Cognitive impairments associated with ASD are nowadays well documented. They include impairment in neurocognition (mainly executive functions) as well as SC (3, 4).

The concept of “executive function” (EF) refers to the higher order control processes necessary to guide behavior in a constantly changing environment (5). This concept encompasses a range of abilities such as planning, working memory (WM), mental flexibility, response initiation, response inhibition, impulse control and monitoring of action.

Regarding social cognition, it's a complex construct that refers to the perception and interpretation of social information. It includes several domains such are social perception, emotion recognition and theory of mind (ToM) (6).

A systematic review and meta-analysis (including 75 studies) investigated the patterns of non-social and social cognitive functioning in patients with ASD. Results showed that the ASD group, compared to neurotypical adults, exhibited impairments across all domains of non-social and social cognitive functioning, with the largest deficits in social cognition (ToM and emotion perception and processing). Regarding non-social cognition, the most impaired domains were processing speed, verbal learning, memory, reasoning and problem solving (4).

Given the tremendous impact of these alterations on everyday life, appropriate interventions are required (7).

In this context, cognitive remediation (CR) is an innovative therapeutic approach which has known an important gain of interest over the last decades (8). It's a kind of rehabilitation treatment aiming to reduce cognitive deficits through repetitive exercises and positive reinforcers (9). CR has been widely studied in patients with schizophrenia. The efficacy of integrated approaches combining social and non-social remediation has been supported by several studies with regard to this condition (10, 11).

Although CR studies in children and adolescents with ASD are still new, the available evidence suggests that it could be a potential efficient approach (12). Research studies involving CR interventions targeting both neurocognitive and social cognition are lacking.

The present perspective aimed to highlight CR programs contribution in ASD (especially in children and adolescents), and then to discuss the interest of combining social and non-social programs, in light of their effectiveness in schizophrenia, given the overlap between these two conditions.

## Effects of CR in children and adolescents with ASD

Initially used for brain-damaged patients, CR use was extended to schizophrenia (13), and later to several pediatric mental health conditions. CR effectiveness was demonstrated in children and adolescents with attention deficit hyperactivity disorder (ADHD) (14), anorexia nervosa (15, 16), specific learning disorder (17), intellectual disability (18) and in early onset psychosis (19).

Literature data regarding CR in ASD is still young, especially those involving children and adolescents. Dandil et al. conducted a systematic review presenting all peer-reviewed published studies of CR approaches in patients with ASD (7). The 13 studies included in this review are divided into four randomized control trials (RCTs); two non-randomized control trials, four case series, two feasibility studies and one case study.

Among these 13 studies, only four involved children and adolescent, two of them were carried out in Children and Adolescent Psychiatric Department in Razi Hospital in Tunisia. It was a cross-sectional study including 25 children and adolescents with ASD. The implemented CR program was CRT (Cognitive Remediation Therapy). It is an individual treatment using pencil and paper tasks and relying on cognitive strategy instruction. CRT program applies novel techniques such as errorless learning and scaffolding. It comprises three modules: cognitive flexibility, working memory and planning (20). The CRT program was provided at the rate of one session per week. The evaluated parameters were intellectual abilities, cognitive flexibility, inhibition, WM, planning, clinical symptoms and school results. Among the 25 subjects included, 16 completed the program. After CRT, children showed significant improvement in intellectual abilities (p < 10^−3^), scores of phonemic flexibility (p = 0.027), WM (p = 0.003 for the forward digit-span and p = 0.003 for the backward digit-span), clinical symptoms (*p* < 10^−3^) and school results (*p* = 0.001) (21, 22).

Social cognitive measures weren't included. Nonetheless, interestingly (and unexpectedly), a positive indirect effect of CRT was observed on clinical symptoms. This improvement could suggest a possible (but difficult to confirm) effect on social abilities. The assumption is that improvement on EF could lead to generalization to global functioning (13).

In order to ensure these effects durability, monthly coaching sessions were provided. During these sessions, therapist verifies that participant correctly applies learned strategies in everyday situations.

In another study (RCT), 121 children with ASD, aged from eight to 12, were randomly assigned to an adaptive WM training, an adaptive cognitive flexibility-training, or a non-adaptive control training (mock-training). All children in all conditions improved in WM, flexibility, attention, and parent rated EF, social behavior, ADHD-behavior, but not in inhibition. Hence, the lack of differential improvement between the adaptive training condition and the mock one suggest that this intervention (in its current presentation) is not suitable for children with ASD (23).

It's worth noting that in theseabove-mentioned studies involving children and adolescents (22, 23) only programs training EF were used.

Five studies included adults with ASD, one didn't find any improvement (24). One other study showed no significant improvement made by CR (25).

In three studies (3, 26, 27), only social cognition training programs were implemented. All of the results showed significant improvement. This improvement was observed on facial affect according to Bölte et al. (3), face and voice recognition measures (26), or ToM and social cognition skills (27).

Only two studies conducted by Eack and co-workers, investigated an integrating treatment approach targeting both non-social and social cognition (28, 29). This program called Cognitive Enhancement Therapy (CET) made a significant improvement on neurocognitive performance and social cognition measures.

Another interesting recent systematic review carried out by Pasqualotto et al. in 2021 investigated the effectiveness of EF studies in children and young people (up to 23) with ASD (12). Nineteen studies were included, involving either computerized or non-computerized trainings. As for non-computerized programs, results showed an overall improvement on EF (mainly on cognitive flexibility, problem solving). Regarding computerized approaches, there was a significant improvement on some EF specifically attention, WM, and inhibitory control.

Certainly, the results of these literature reviews bring some clarity regarding CR interventions in ASD, as much as these programs are potentially effective (7). Nonetheless, these studies also raise questions related to their generalizability, and conditions enabling to reach an optimal level of effectiveness.

## Relationship between social cognition and EF

A large body of evidence has supported the association between executive processes and social cognition abilities (12). The latter require, among other factors, self-mentoring skills and response initiation, which are impaired in patients with ASD (30).

Moreover, self-awareness and mentalizing require some EF, mainly monitoring actions and acting with volition. Hence, impaired EF were suggested to weaken theory of mind (ToM) abilities (31). It's worth noting that the latter ability is the social cognitive component that has received most interest and attention. Its daily life application requires a certain number of involved EF such as inhibition, WN, attention (32).

Subsequently, in a study conducted by Jones et al. (31) in 2017, results yielded that mentalizing difficulties were associated with more severe social communication symptoms in adolescents with ASD.

A part from ToM, processing social information when interacting with other people requires undoubtedly intact WM. Likewise, engagement in social interactions needs also mental flexibility, planning as well as generating novel thoughts or actions and finding various alternatives to solve little problems related to relationships challenges (1).

In line with all these findings, research conducted by Miranda et al. (32), in 2017, investigating the relationship between EF and social cognition in high-functioning ASD, concluded to a strong association between social cognition and metacognitive processes such as initiation and planning.

In addition to that, it has been strongly suggested that EF could underlie ASD core symptoms. In fact, repetitive behaviors could be underpinned by impaired mental flexibility.

Moreover, another argument related to neuroanatomy, is that brain structures engaged in social cognitive processes comprise areas involved in EF (prefrontal cortex) (33).

At last, and importantly, results from some research studies in schizophrenia support the hypothesis that intact neurocognition could be considered as a “necessary precursor” for adequate social cognition (34). Hence, results reflected in all these studies support our hypothesis about combining social and non-social remediation in the management of ASD. Indeed, when coupled with CR interventions focused on EF, the benefits from remediating SC would be greater. Further than that, these findings provide strong evidence to suggest that EF should be trained first.

## Integrative therapeutic approach for ASD

Unfortunately, studies regarding CR approaches combining social and non-social remediation are scares. Typically, in literature studies, only one of these two domains is trained with little or no emphasis on the other (35).

In fact, a meta-analysis performed by Fletcher-Watson et al. (36), investigated the efficacy of ToM interventions in subjects with ASD. Authors concluded that even if ToM was trained, there was a little evidence of generalization beyond task performance to other settings or related abilities.

Besides, in studies using CRT, it is worth noting that some exercises could indirectly train ToM, such as ≪ Figure-ground ≫, in which participants have to consider different points of view (or different perspectives), and then to recognize that one's mind is different from others mind. Moreover, generalization to everyday life is ensured by homework tasks. Despite all of this, during coaching sessions, we still observe difficulties in social abilities. In fact, participants still display impairments in abstract abilities, initiating conversations and language pragmatic.

Therefore, all aforesaid findings could lead to implications for cognitive interventions in ASD. In fact, training an isolated aspect of cognition is likely not sufficient, because it is actually hard to draw conclusions about its generalization. Hence, therapeutic remediation modalities associating both domains of cognition could lead to interesting results, especially since integrative approach has been beneficial in schizophrenia. Indeed, results obtained from studies involving integrated interventions on neurocognition and SC showed larger effects on some neuro- and social-cognitive domains, symptoms, and functional outcome compared with treatment as usual (11). Several interventions were used such as REHACOP, integrated neurocognitive therapy (INT), integrated psychological therapy (IPT) and cognitive enhancement therapy (CET) (37–39). Besides, as previously stated, the importance of remediating neurocognition as a prerequisite for social cognition has been strongly suggested (34).

Regarding integrative CR approach in ASD, only two studies were carried out [by Eack et al. (28, 29) in 2013 and 2018]. They sought to evaluate the efficacy of CET in improving neurocognitive and social-cognition variables, in ASD adults. CET is an 18-month multidimensional program integrating neurocognitive training targeting attention, memory and problem solving, as well as social cognitive training. The latter focuses on taking other people's perspectives in order to address social context appraisal, reciprocity and affect regulation (35). Neurocognitive sessions and social-cognition training were held concurrently. Overall, results showed significant improvement on neurocognition outcomes (versus enriched supportive therapy). As for social cognition measures, significant advantage was found at nine but not at 18 months.

CET offers several advantages such as mutual support and encouragement between participants during group exercises (37). According to Hogarty et al. who applied CET for schizophrenia patients before its adaptation to ASD population, improvement in processing seemed to be the principal mediator of CET effects.

These findings underscore the benefits of combining neurocognitive and social cognition remediation for patients with ASD. Furthermore, they support the need for early CR interventions (7).

Furthermore, training social skills would be beneficial for these patients. Indeed, this would allow a more efficient transfer to everyday life of the knowledge acquired during the integrated remediation (neuro and social cognition). Psychosocial rehabilitation promotes personal recovery, successful community integration and satisfactory quality of life for persons with mental illness. Based on specific therapeutic tools like CR, psychoeducation, behavioral and cognitive therapy and social skills training (SST), it has shown its effectiveness in the treatment of ASD (40). In this context, many intervention studies using several SST programs such as Social Cognition and Interaction Training (SCIT), UCLA Programme for the Education and Enrichment of Relational Skills (PEERS), Assistive Soft Skills, and Employment Training (ASSET) have been carried out (41).

SST refers to a wide range of group-based interventions and instructional methods commonly used in individuals with ASD to help understand and improve social skills (42).

A recent metanalysis of controlled studies has been conducted to evaluate the effectiveness of SST in adults with ASD. Better improvement in social responsiveness was shown in participants included in the treatment than those in control groups (43).

## Concluding thoughts

Being a major source of disability in patients with ASD, cognitive deficits should represent a linchpin around which therapeutic interventions revolve.

CR is a promising therapeutic approach. The increasing awareness of its potential effectiveness in managing several mental health conditions has fostered considerable interest in the area of ASD. Although literature on CR in ASD is still young, the available evidence suggests that it could be a beneficial approach.

There is a paucity of studies that have involved programs targeting both neurocognitive and social cognition. Since the majority of studies have focused on training one aspect of cognition, it remains unclear whether the effects might be generalized to other aspect. Only in two studies using an integrative program named CET, effectively led to improve cognition among this population.

Despite the fact that this perspective is not based on a systematic approach, combining social and non-social CR approaches could be interesting in ASD. In order to firmly recommend this integrative approach, further studies are required. Besides, studies including larger samples are needed to elucidate its efficacy and understand predictors of attrition and efficiency. In this context, increasing patients' motivation by implementing more digital technology programs currently widespread could be attractive (39). Furthermore, we believe that implementing such programs within the school setting could lead to a greater impact on their overall functioning.

## Data availability statement

The original contributions presented in the study are included in the article/supplementary material, further inquiries can be directed to the corresponding author.

## Author contributions

MH, ZA, HY, SJ, SH, and AB conceived this perspective. MH authored the first version of this article, which was then revised and optimized by ZA, AM, and AB. All authors contributed to the article and approved the submitted version.

## Conflict of interest

The authors declare that the research was conducted in the absence of any commercial or financial relationships that could be construed as a potential conflict of interest.

## Publisher's note

All claims expressed in this article are solely those of the authors and do not necessarily represent those of their affiliated organizations, or those of the publisher, the editors and the reviewers. Any product that may be evaluated in this article, or claim that may be made by its manufacturer, is not guaranteed or endorsed by the publisher.
